# Modification of Nursing Education for Upgrading Nurses’ Participation: A Thematic Analysis

**DOI:** 10.5539/gjhs.v7n4p161

**Published:** 2014-12-31

**Authors:** Akram Aarabi, Mohammad Ali Cheraghi, Shahrzad Ghiyasvandian

**Affiliations:** 1 School of Nursing & Midwifery, Iran University of Medical Sciences, Tehran, IR Iran; 2 School of Nursing & Midwifery, Tehran University of Medical Sciences, Tehran, IR Iran

**Keywords:** nursing education, educational policy, participation, Iran, nurse leader, thematic analysis

## Abstract

**Background:**

The product of the educational nursing programs in Iran is training nurses who less have professional apprehension and commitment for participating in professional decisions. Whereas nurses especially those in high academic levels are expected to more involve in professional issues.

**Objective:**

The aim of this study was to explore Iranian nurse leaders’ experiences of making educational nursing policy with emphasizes on enhancement of nurses’ participation in professional decisions.

**Methods:**

We used a qualitative design with thematic analysis approach for data gathering and data analysis. Using purposive sampling we selected 17 experienced nurses in education and making educational nursing policies. Data gathered by open deep semi-structured face to face interviews. We followed six steps of Braun and Clarke for data analysis.

**Results:**

In order to enhance nurses’ participation in professional decisions they need to be well educated and trained to participate in community and meet community needs. The three main themes that evolved from analysis included opportunities available for training undergraduate students, challenges for PhD nurses and general deficiencies in nursing education. The second theme includes three sub-themes; namely, the PhD curriculum, PhD nurses’ attitudes and PhD nurses’ performance.

**Conclusions:**

We need for revising and directing nursing education toward service learning, community based need programs such as diabetes and driving accidents and also totally application of present educational opportunities. The specialization of nursing and the establishment of specialized nursing associations, the emphasis on teaching the science of care and reinforcing the sense of appreciation of pioneers of nursing in Iran are among the directions offered in the present study

## 1. Introduction

Education has a prominent role in bringing about changes and obtaining anticipated results. Education is a basic means to get ends. Many useful changes and transformations had been possible by developing appropriate educational backgrounds. Nurses need to participate in decisions in order to create a better job environment and better care of their patients. But their participation is not adequately accountable (Ressler & Glazer, 2011). The history of nursing education is related to the history of nursing policy making. During years by developing educational and practical nursing standards through effective policies by nurses, this thought came to minds that nurses are able to self regulation and self direction (Gittens-Scott, 2008). Nightingale believed that nursing affairs should be controlled by nurses. Her writings which had been gathered in 1916 presents her knowledge about policy making. Acceptance of her beliefs in all over the world about health and separation of the essence of nursing discipline from medicine well display her ability in making policies and changes (Mason, Leavitt, & Chaffee, 2011).

In recent years nursing education in Iran has considerably developed. Following expansion of various specialties in medicine and establishment of educational planning committees for each medical specialty in Iran’s ministry of health and medical education (MHME), nursing educational planning committee also established and its status in MHME stabilized. This committee is the most elucidate unite for nursing educational policies in Iran (Vaismoradi & Parsayekta, 2011). Nowadays nursing educational policies are planned in the Bachelor of Science level which is the lowest level of academic nursing in Iran, also in the Master of Sciences level with eight approaches and finally in the Philosophy Degree (PhD) level. But since nursing education transferred to nursing faculties, nurses often have been criticized for deficiency in performing suitable education for improvement quality of patient care (Hajbagheri & Salsali, 2005) and transferring theoretical knowledge to practice. Then in spite of improvement in nursing academic educational development, still nurses’ behaviors are based on traditional approaches (Cheraghi, Salsali, & Safari, 2010). Also the other products of this approach is training nurses who less have professional apprehension and commitment for participating in professional decisions. Whereas nurses especially those in high academic levels are expected to more involve in professional issues (Milsted, 2008).

Participation in lexicon means to have a share, to attend as a member and to partner together. Boswel, Cannon and Miller (2005) have defined participation in policy making as presentation of activities and behaviors for effecting on legislation and governmental strategies. Nurses’ participation in policy making is effected by nursing education (Arabi, Rafii, Cheraghi, & Ghiyasvandian, 2014). This effect is considerable from two aspects. The first is that nursing education in both graduate and undergraduate level should be designed by a way to directly increase nurses’ knowledge about policies. In this regard International Council of Nurses (ICN) has stated that nurses should have enough knowledge about how policies are made and implemented, because without elucidate perception of how policies are made and implemented they can not have effective participation in policy making process (Milsted, 2008). The second aspect is that the special style of nursing education as a background will lead toward training nurses who enjoy more professional development and more involvement in different levels of policy making for nursing. In this regard Boswel et al. (2005) nominated three levels for nurses’ participation in making policies include: presentation, success and dominance. In presentation level, nurses as novice arrive in the world of policies. This is a minimum point for nurses’ growth. In this level nurses participate in society servicing and in voting. Some nurses stay at this level until the end of their professional lives and of course this is an appropriate level of participation for them. As nurses’ social health knowledge and commitment enhance, they select entrance to the second level which is success. In this level nurses sensitize to inequalities, challenges and agendas related to health care. These nurses usually are candidate to speak from the side of a group or a representative and/or to give advises. The last level is dominance. In this level provident nurses deeply involve in development and promotion of health policies.

however as mentioned, nurses’ education and nurses’ participation in making policies have some relationship, but we do not have a clear insight about the points of nursing education and domains of nurses participation in Iranian context. The aim of this study was to explore Iranian nurse leaders’ experiences of making educational nursing policy with emphasizes on enhancement of nurses’ participation in professional decisions. This study is part of a broad study about the outputs of nursing educational policies.

## 2. Method

### 2.1 Design

In this study we used a qualitative design with thematic analysis approach for data gathering and data analysis. Interpretation is inevitable part of qualitative research but the essence of interpretation in qualitative researches is different based on the approaches employed. In grounded theory and hermeneutic phenomenology the interpretative essence is the most, because in these approaches researchers deeply follow the process and context of the subject under study (Sandelowsky, 2010). When it is not possible for researcher to deeply clarify the process and the context of the phenomenon under study, it is common to employ qualitative descriptive approaches like thematic analysis which enjoy less interpretation essence. Thematic analysis is a principle method for qualitative data analysis through discovering, analyzing and reporting themes which have been emerged from data. In this approach researcher make a rich description of the subject and also interpret different aspects of it (Braun & Clarke, 2006). By this way thematic analysis is separable from qualitative content analysis which is more dependent to frequencies of codes for data categorization and has less interpretation essence (Vaismoradi, Turunen, & Bondas, 2013).

### 2.2 Participants

We used purposive sampling and selected 17 experienced nurses in education and making educational nursing policies. Inclusion criteria were having a history of nursing education and also a history of nursing management in various managerial levels. In order to have a maximum variation sampling, participants were selected among members of the Iran’s board of nursing, dean of nursing faculties, members of the supreme council and the boards of directors in Iranian Nursing Organization (INO), heads of the Iranian Nursing Association and the Iranian Scientific Nursing Association and also matrons with the history of nursing education in past and present. Of them six were female, 11 were male, two had bachelor degree, four had Master of Science and the rest had PhD in nursing education.

### 2.3 Data Gathering

Data gathered by open deep semi-structured face to face interviews. The time for each interview was 60 to 80 minute. The first author carried on all interviews. First the interviewer called to participants or their secretaries, introduced herself, gave some information about the purpose of getting into contact and finally requested for an appointment. All interviews took place in a calm environment in participants’ offices. In order to increase credibility and dependability of data, all interviews begin with this key question: As a nurse educator leader please share your experiences about the portion of educational nursing policies in training nurses who have more willingness to participate in professional decisions. Meanwhile to get more clear responses, we used statements such as why, how, and so on to probe answers.

### 2.4 Data Analysis

We followed six steps of Braun and Clarke, (2006) for data analysis. The first step was acquainting with data. In this step the content of recorded interviews were transcribed verbatim. Each interview read for several time and noted about ideas. The second step was initial coding. In this step interest features among participants’ statements coded thoroughly and in accordance to whole data. By this way relevant data appeared. The third step was to look for themes. In this step similar codes put together resulted in initial themes. The forth step was reviewing themes. In this step temptation carried on to confirm coordination between codes, related initial themes, and the entire data set. By this way a thematic map generated. The fifth step was definition and naming extracted themes. In this step the entire process of analyzing reviewed to refining the characteristics of each code and the story which the whole analyzing process tells. Then each theme clearly named and defined. The sixth and the last step were reporting. In this step we returned to research question and after adjustment, results were prepared for reportage. All interviews and initial coding carried on by the first author and the second and the third author revised coding process, extracted themes and refined data. We negotiated and discussed about the way of coding and extracting themes frequently until agreement achieved.

### 2.5 Rigor

Qualitative data should have as much trustworthiness as possible (Speziale, Straubert, & Carpenter, 2007). In order to attain credibility, researchers spent enough time for emerging data and also did member checking. For this the first author gave transcribed interviews to related participants and then corrected the initial codes according to their opinions. We also did peer checking by using opinions of two of our coworkers who were well experienced in qualitative and thematic analysis for coding and extracting final themes. For enhancement of dependability we tried to do all interviews in a range of certain time and also used audit trail and explained the entire research stages step by step.

## 3. Results

Findings of the present study are reported within three main themes; that is, opportunities available for training undergraduate students, challenges for PhD nurses and general deficiencies in nursing education. The second theme includes three sub-themes; namely, PhD curriculum, PhD nurses’ attitudes and PhD nurses’ performance. The following interpretations demonstrate the presented themes along with direct quotes from participants.

### 3.1 Opportunities Available for Training Undergraduate Students

Nursing education is currently composed of hospital training. However, hospitals alone cannot properly display nurses’ capabilities for care; it is therefore crucial to extend the scope of nursing education and performance to the community. Nurses who enter the social arena from the outset are directly exposed to the community’s health demands and become more inclined toward participation. Participants believed that there are plenty of opportunities ahead of them that have been little explored so far. Introducing the capabilities of nurses to the community, especially with the assistance of nursing students, is one such opportunity that some respondents referred to are as follow:

*“For example, today is the World Diabetes Day. Go to the subway station, places where there are plenty of people, and set up a stand, for people to gather around, and teach them about diabetes. You’ll see how many people will gather around for them” (former Nursing Board member)*.

*“Considering the high rate of traffic accidents in this country, we should set up an office composed of students for teaching traffic rules on the go” (Board of Directors member, INO)*.

Another issue that was of interest to the participants is the use of opportunities for role-model setting in nursing. In fact, one of the best ways for teaching nursing is through observation and through helping the students choose role-models from among teachers who have been influential on the nursing profession. Few respondents referred to this issue. The following is an example:

*“From the day a nursing student, who used to be a high school student just until now, sets foot into nursing school, her first model is the teacher. So, their teacher should be one who is outstanding, more so for new students” (dean of faculty and former Nursing Board member)*.

Participants proposed nursing literature as a source of coming up with ideas for increasing nurses’ involvement in the community and encouraging their participation in professional decision-making in care, and believed that this way, nursing regains its professional identity in care. A few participants said:

*“To whom does palliative care belong? Its basis belongs to nursing folks. Where did you first see this particular literature on elderly care? They all came out of nursing” (Board of Directors member, INO)*.

*“We have strong resources for nursing. Other disciplines have used them and have gained an identity for themselves through them. The resources belong to us and yet we have gained no identity out of them” (dean of faculty)*.

### 3.2 Challenges for PhD Nurses

Educating PhD nurses has dramatically increased in the past few years. The increased number of PhD nurses has provided them with the opportunity to experience a greater presence in higher positions of decision-making with an increased executive power. However, there are still many criticisms about PhD nurses’ inadequate empowerment for getting more effectively involved in benefiting from all available capacities. The present study classifies these criticisms under 3 sub-themes; namely, PhD curriculum, PhD Nurses’ attitudes, and PhD nurses’ performance. Findings of the study along with pertinent quotes follow.

#### 3.2.1 PhD Curriculum

According to participants, the PhD courses available are not consistent with the demands of the Iranian community. In PhD courses, we need nurses with specialized capabilities in various fields in order to meet the demands of the community. The examples are cancer, diabetes, renal, and trauma nurse. Direct references to the need for a more specialized PhD course are presented by numerous respondents as in the following quotes.

*“Well, a student might have specialized in something for her master’s, in elderly care for example. Now we can let her work at the related areas, and we make her/his to pass numbers of courses, seven, eight, ten courses. This becomes a PhD with specialization in elderly care. Then, we let her loose in the ward, and hopefully she becomes an expert in these diseases. Or, she can take part in policymaking for the elderly”. (Nursing Board member)*.

*“…A bachelor’s degree suffices for our nurses in the surgery department. But we have the cardiac surgery department, and then the pediatric cardiac surgery department, as subspecialty… We may need a specialist there who knows both pediatric nursing and pediatric cardiology nursing. She should know neonatal care, and be able to manage the work. That’s what a clinical nurse specialist is.” (former Nursing Board member)*.

Nursing specialization at the PhD level smooth the way for the establishment of specialized nursing associations according to the demands of the community. Some respondents quoted:

*“We have so many PhD graduates in nursing education. So a single association suffices. When you have a PhD in pediatric nursing, then they say it’s a specialization, and you will be given a permit for an association” (former dean of faculty)*.

*“When we have a specialized nursing association, our participation will expand. When people see on a board the words “specialized renal care nursing association”, then they’ll come visit it. All specializations in nursing can have an academic association. These are the things that show our worth and value to the community” (former Nursing Board member)*.

The need to strengthen management and leadership skills of PhD students through special or complementary courses so as to increase leadership capabilities and participation in decision-making was another issue raised by a few of respondents as follow:

*“Perhaps we need to plan specific courses for these people, or we should send them away for specialized trainings” (head of a Nursing Association)*.

*“It means that you should be able to negotiate and interact with those people. You should be able to score points. Not everyone has these attributes; only special people do. But these attributes can be strengthened through special courses” (former Nursing Board member)*

#### 3.2.2 PhD Nurses’ Attitudes

Participants believed that many PhD students or graduates did not have a befitting attitude toward the field of nursing and patient care. The attitudes of PhD students were criticized from several angles. The present study mainly criticized how it is neglected by PhD students or graduates that the patient is the focus of the delivery of services, and how the mission and the goals of the field of nursing in providing patient care are also forgotten. Some respondents quoted:

*“If you come to one of these brainstorming sessions of PhD students in nursing, then you’ll see where the patient’s position is in this set-up!” (head of a Nursing Association)*.

*“We don’t see the patient, we see ourselves. The education system does not see the patient. Education is merely writing papers, doing research and the like. We will be in trouble if we ever let go of the patient, because we are here to serve the patient; and we have forgotten that” (former Nursing Board member)*.

The tendency to avoid the title of “Nurse” by PhD graduates was another point for criticism. Some respondents especially those who did not have PhD degree stated:

*“We go through so much trouble trying to prepare a program for the National Television. We search everywhere for someone who has something to say in this field, and it is about to go on air. Then, he says “don’t refer to me as a nurse, write, assistant professor of so and so university. Can a person with such an attitude make policies for nursing? This is such a big issue” (Supreme Council member, INO)*.

*If you were in a session and did not introduce yourself as a nurse,…then how you can make policies for nursing (former Nursing Board member)*.

#### 3.2.3 PhD Nurses’ Performance

Participants believed that nursing policymakers’ recent attaining of the PhD title has been a major opportunity for them, because this title carries a positive weight in the society and can help the nurses’ performance in the community and the acceptance of their views in decision-making meetings. In other words, this title enables them to have their plans and ideas accepted by the authorities. This is illustrated by many respondents as in the following quotes:

*“They say that Picasso used to leave the gallery doors at his exhibitions wide open. When asked, ‘why? They may steal these works”, he says, ‘never mind as they are not signed. My works are only valuable when I sign them’. After all, titles play a role, right or wrong, I’m not here to judge. When you have the PhD title, it helps you defend nursing rights and patient rights much better. Now how much we use this” (former dean of faculty)*.

*“Degrees comprise a major value in our present culture… The question is, how much does these degrees serve to the health of the community?” (Nursing Board member)*.

Many respondents believed that the poor practical skills of nursing instructors, especially those with PhDs, has reduced the level of trust and has distorted nursing education as stated by respondents in the following quotes:

*“Knowing your job generates trust. Trust leads to communication, and gradually, one finds her place in the society. Now how trustworthy are these people who take to teaching?” (former Nursing Board member)*.

*“How much practical prowess do I have as an instructor? How close is our instructor to the patient? What do we do when they say, ‘get the patient off the bed after surgery’?! … This is how we expose ourselves to the public” (former dean of faculty)*.

One weakness of PhD nurses’ performance in policy making positions is making clinical policies without sufficient knowledge of clinical work and patient safety. Some respondents said:

*“Our weakness right now is when we want to make clinical plans, but our policymakers are mostly having an educational view and are less familiar with clinical procedures. Clinical plans should be devised by someone who knows the issues with clinical practice.” (head of a Nursing Association)*.

*“In our planning, we should first and foremost be focused on the safety of people receiving services; only then should we contemplate how nursing education should be conducted to ensure this safety” (matron with 28 years of experience)*.

### 3.3 General Deficiencies in Nursing Education

General deficiencies in nursing education policies in our country are those which not specific to any particular course and degree; rather, these deficiencies affect the entirety of the nursing education system and the training of nurses participating in nursing policymaking. Participants’ quotes regarding this issue follow.

The biomedical approach to nursing education is considered a barrier to training nurses participating in society’s health issues. Being convinced by the fact that nurses have a particular knowledge of care that other medical team members lack, increases the chances for their participation. Some respondents shared:

*“There is a problem with our education. It does not reinforce specialized nursing knowledge; rather, it simply conveys medical knowledge. Care knowledge is not reinforced as a result. That is why one thinks she does not have specialized knowledge and expresses her opinions to a lesser degree” (Board of Directors member, INO)*.

*“The condition of nursing in our country is such that a graduate has touched on everything, but does not know anything, especially in her own field, which is the science of care. Now, when at a clinical setting, she wants to show herself, still a physician knows several times as much, so she has nothing to offer. We have not taught her anything called the science of care that can be a source of power for her and enable her to get involved” (Supreme Council member, INO)*.

Poor feedback from the community with regard to educational policymaking was another deficiency in nursing education. Some respondent stated:

*“When we don’t know the expectations of the community from nursing, how are we supposed to educate students? How much do we seek information that helps us plan for the demands of our community?” (matron with 25 years of experience)*.

*“Does all this planning result in meeting our expectations of nursing?” (former Nursing Board member)*.

Also poor coordination between nursing policymaker institutions is indicative of these deficiencies. Many respondents quoted:

*“Education is doing its job. It trains the workforce. That’s about it. What does this trained workforce do at a clinical setting? What is she meant to do in the community? Nursing has not been contemplated holistically. Education does its own job, and clinical practice also its own” (matron with 25 years of experience)*.

*“When we do one thing bit by bit, and these bits are unrelated, they may neutralize each other. That is not policymaking. Policymaking finds its meaning within a system. Nursing training is distorted this way” (Nursing Board member and dean of faculty)*.

Recognition and appreciation of pioneers of nursing and available positions in policymaking is another issue less clarified for students of nursing. In fact, strengthening the sense of recognition and appreciation in students reinforces their desire to participate. A few respondents shared:

*“Faculty members keep saying ridiculously, what did the Iranian Nursing Organization achieve? I want to say what did you want it to achieve?” (Nursing Board member)*.

*“It would be wonderful if we thought about the pioneers of our profession, if we knew what they have gone through for us today to have this position. Nurses should know those who have served their profession both in the past and in the present day” (former Nursing Board member)*.

Unequal opportunities of clinical growth for physicians and nurses were considered a condition of our health system that has hindered the development of the practical capabilities of other groups, including nurses. Therefore, they cannot have an effective participation in care-related issues. However that was not experienced by all respondents. A few respondents stated:

*“When you expand liver transplant, or whatever, without having any especial care training for your nurses, then how can you expect a nurse with little preparations and little motivation to care for that patient?” (Nursing Board member and dean of faculty)*.

*“We should all progress together, all of us. Others should have equal rights as physicians to grow, so that they can grow as well” (matron with 25 years of experience)*.

At the university level, medical schools are usually the center of attention in general policymaking and form another condition governing our health system. However a few respondents belived that this condition did not have a considerable effect. Some congruent respondents quoted:

*“Once, in a meeting with the head of the university, I made the analogy of a hydrocephalus kid for comparing medical schools to other schools; this kid has a huge head, that is the medical school, but he has a weak body, which is other schools. This kid won’t be able to walk” (Nursing Board member and dean of faculty)*.

The non-academic view of some physicians on the field of nursing has made conditions for the participation of nurses in decision-making more difficult. Some respondents said:

*The top down vision of some physicians to nurses as less knowledge persons obstruct having their* *opinions (Supreme Council member, INO)*.

*“Other than 24-hour patient care, what do our physicians expect of nurses? Do they consult nurses about care issues and improving patients’ health? Do they accept that nurses also possess the required knowledge?” (former dean of faculty)*.

**Table 1:** Participants’ demographic characteristics

Managerial status	Number	Mean years of management experience
Dean of faculties	5	6.8
Nursing Board members	5	22.47
Iranian nursing organization supreme council	3	6.03
Iranian nursing organization boards of directors	2	2.93
Heads of Iranian nursing associations	2	13.71
Matrons	4	25.2

*Note*. Some of participants had more than one managerial status, so the sum of numbers is more than 17.

## 4. Discussion

The first theme of the present study was the opportunities available for training undergraduate students. Our instructors have not properly used available opportunities for providing better community services with regard to diabetes education and prevention or to present practical projects in accordance with the rich literature available on nursing and the recent scientific advances of the world. This mode of learning, known as service learning, prepares nursing students for greater, better participation in the community (Emerson, 2007). Opportunities for community presence thus provide new students with the chance to practice focusing on the patients’ concerns and to understand the impact of nursing services on the health of the community (Hughes, 2002). A sample of service learning is conducted by Byrd et al. (2012). Following an active learning experience on the subject of community health nursing and participation in related activities, 300 undergraduate nursing students from Iceland were requested to complete a questionnaire determining the level of political intelligence. Results obtained showed a significant increase in the students’ mean score of political intelligence following participation in these learning activities (P=0.0). Boswell et al. (2005) have proposed the framework of nursing curriculum as a huge barrier to the participation of nurses in nursing policymaking processes, which particularly keeps nurses away from social activities and community healthcare services. Conger & Johnson (2000) suggest the deficiencies in nursing education to be the shortage of courses related to community health nursing at the undergraduate level.

Making use of available opportunities for training undergraduate students is a part of the educational policymaking process. The process of policymaking is influenced by three moves, including, the alignment of problems, the identification of possible solutions and favorable political environment that accepts the problems and solutions. It is vital to notice the opening of the windows of opportunity in all three moves (Hewison, 2008). Ferguson (2001) argues that the opening of the windows of opportunity does not mean for us to just sit and wait for the windows to open some day and provide us with an opportunity; rather, the history of our involvement in the community, our familiarity with the problems and the community’s expectations of nursing, and finally having potential solutions at hand all facilitate the opening of the windows of opportunity.

The second theme of the present study is composed of challenges for PhD nurses. The first sub-theme challenges nursing PhD curriculum and proposes the specialization of nursing at the PhD level and also the development of specialized nursing associations. Looking at other PhD curriculum, we see that this is offered both with a specialization in nursing education (as is the case in Iran) and with other fields of specialization based on demands of the community. For instance, at the University of Nevada in the United States, nursing PhD is currently offered with two specializations –one in education and another in civil support. The civil support specialization has been designed with the purpose of training specialist nurses able to provide specialized community health services (Nevada’s PhD in nursing program, 2011). As for the promotion of specialized nursing associations, there are currently 60 associations, committees, and societies in the United States involved in specialist nursing care activities (Mason et al., 2011). In Iran, there is one active specialized nursing association, called the “Iranian Nursing Cardiac Association”.

This sub-theme also emphasizes increasing course credits with a content of policymaking in the health system in order to acquire multifaceted capabilities and train nurse managers with policymaking abilities especially at the top policymaking levels. It is evident that nurses who have acquired skills and knowledge in areas related to nursing, such as, teaching, research, management, and even areas such as, epidemiology, health economics, etc., have a better chance at entering policymaking fields. With regard to nursing education at the master’s level and the PhD level, Conger and Johnson (2000) write, “there is a shortage of trained nurses with master’s degrees who can assume social responsibilities in the name of nurse leaders in order to further advance the healthcare system, and at the PhD level, there is a shortage of training in policy analysis, research in policymaking fields and models”. Reutter and Duncan (2002) have emphasized the addition of public health policymaking courses for a better understanding of policymaking processes as a major need of preparing nurses at the graduate level.

So far, various programs have been designed with the purpose of preparing nurses or nurse managers for a more effective and active presence in policymaking fields, management and for revision making. In Iran, nurse managers and PhD students can also benefit from these programs in graduate studies. For example, the Leadership For Change (LFC) program is mostly the reinforcement of leadership skills and change management (Benton, 2012), Nevertheless, for gaining more benefit from the LFC program, taking some other courses are also necessary, such as courses in the Management Business Organization (MBO), mini-MBOs, health policymaking, health economics and entrepreneurship. MBO courses are currently being offered in Iran by the MBO Association of Iran for different businesses. We should not forget that these courses do not easily provide us with the prerequisites for entering the field of nursing policymaking; in fact, entering this field requires a series of capabilities, which is only partially reinforced through these courses. On the same note, Hughes (2005) writes, “it is true that policymaking has its own particular theoretical foundations and that we need to boost our brain if we are to advance in policymaking; yet, policymaking is not merely an intellectual activity; more than anything, it is a practical tool for making changes”. Jasper (2002) addresses one of these courses in his article titled “Leadership Empowerment Organization” (LEO), and writes, “although programs like the LEO have strengthened the managerial skills of nurse managers, we cannot expect anyone to be an influential clinical manager just by passing these courses, as this end will only be achieved by using practical opportunities to show leadership prowess. In other words, we have to see if the hats have been altered or the heads as well”.

The next sub-theme was the attitude of PhD students. Years spent on fulfilling PhD credits might have kept some students away from the mission and goals of the discipline of nursing and the purpose of training nursing workforce. This change in beliefs has affected their participation in areas of decision-making for nursing and patient issues. In a study on the preparation of nursing students for future presence in the domain of policymaking, Zauderer et al. (2009) conclude that, “without training students who believe in nursing, conditions will not be provided for the future presence of nurses in the policymaking domains”. They therefore consider strengthening the students’ belief in nursing a prerequisite for preparation programs. Findings of the present study also show that, since faculty members and the educated class of nurses -a class to which nursing policymakers also generally belong- have acquired new titles, they no longer wish to carry the title of “nurse”. Cheraghi et al. (2010) also quote some participants addressing the general tendency to avoid the title of “nurse”, “PhD students think they are not a nurse”.

The next sub-theme is the performance of PhD students and graduates. One issue addressed in the present study is the unhelpful use of the PhD title. In our society, the PhD title can facilitate the acceptance of the nurses’ opinions in decision-making and policymaking sessions. Nevertheless, this title has not greatly resulted in the growth of nurses’ participation in decision-making processes. In a study conducted by Toofany (2005) it is argued that there is a greater expectation of nurses with PhD titles to participate in policymaking debates, since they possess a broader viewpoint. But it seems that, despite their PhD degrees and education, the performance of these nurses has not changed much with regard to policymaking processes. The present study addresses another performance aspect of PhD nursing graduates, that is, the lack of confidence in their practical skills, both in terms of patient safety and role-modeling in nursing. Clinical settings form the first place all nurses walk into as nursing students. The practical skills of PhD nursing graduates often working as nursing instructors can lay the foundation of patient safety and role-model setting in the nursing profession for nursing students. In another study by Cheraghi, Jasper and Vaismoradi (2014) one participant quoted “Nurses with PhD titles only possess theoretical knowledge and therefore cannot participate in clinical functions. There is thus not much hope for them to improve the clinical performance of nurses”. In other study conducted by Cheraghi, Salsali, and Ahmadi (2007). the lack of top-notch instructors for guiding and supervising students, unsuitable role-model setting, shortage of nurses with graduate degrees in clinical settings and the contrast between knowledge and performance in clinical settings have been highlighted.

Policymaking from an educational perspective and the remoteness of policymakers from clinical settings comprised another issue raised by participants of the present study, which is particularly effective in the clinical practicality of the policies and decisions made. Keith (2000), Swanson and Stanton (2013) asserted that wherever nursing leaders were engaged in clinical management, they acquired more benefits from policymaking sessions on clinical issues. A look at the composition of nursing board members at the state level in the United States also shows operational level presence at this position. Although the composition of members depends on state laws, it often includes people from various academic and occupational levels. For instance, state boards are mostly composed of Registered Nurses and Advanced Practice Registered Nurses, and to a lesser extent, Licensed Practical/Vocational Nurses with lower academic degrees and occasionally Consumers or patients as well (Russell, 2012). Therefore, clinical nurses with novel, practical ideas for bringing about change to the structure of nursing can also be periodically invited to participate in the Iranian nursing policymaking sessions.

The third theme was general deficiencies in nursing education. A large part of these deficiencies pertain to the foundation of nursing education in our country. In Iran, nursing education does not provide the conditions in which nurses are trained in a way that they acquire primary capabilities for an active presence in decision-making domains. One of the most crucial capabilities that should be conveyed to the students through nursing education is skillfulness and specialization in the science of care, by means of which nurses can appear in nursing policymaking domains of the health system with greater dignity as specialists in the area of care. The present study considered the existence of bits and pieces from the biomedical education system deep within nursing education as responsible for the failure to properly transfer the knowledge of care to students. The educational method used for nursing education has been modeled after medical education and is based on teaching diseases rather than care issues caused by the disease. Dinmohammadi, Payrovy and Mehrdad (2014) write, “Since the third decade of the 19^th^ century, physicians established nursing programs to provide services to physicians in treating patients. This medical training model mostly emphasizes nursing duties and techniques rather than the process of care. The obvious outcome of this role-model setting is fostering obedience in nurses. Such nurses will rarely be able to participate in professional decision-makings by applying critical thinking”. According to Buerhaus and Needleman (2002), an impediment to the participation of nurses in clinical decision-makings is the educational policy in which nursing education follows the role of medical education. They assert that nurses should themselves be the founders of theories and methods for improving the quality of nursing education. Cheraghi et al. (2010) also point out the biomedical educational structure in nursing instructors’ method of teaching. Quoting a nursing student, they write, “teachers allocate a large proportion of a two-hour session of class to explaining and emphasizing diseases and their pathophysiology and mostly convey medical information, so that when they switch their focus to topics of nursing care, the students are no longer in the mood to listen”.

Poor community feedback on nursing policymaking was proposed by the present study as another deficiency in nursing education. When nursing education policies reflect the health needs of the community, they will appear more prominently on the agenda of macro policymakers. One of the most effective strategies for advancing educational policymaking in nursing is therefore to take account of groups that are directly affected by such policies (Canadian Nurses Association, 2010).

In another study, Villeneuve (2008) writes, “efforts have frequently been made to obtain something of what we desired from different areas of healthcare; whereas the right path is for the people we serve and the needs they have to guide us in our achievements”.

Reinforcing the sense of appreciation of pioneers in nursing and the achievements of nursing students, and also the introduction of these pioneers as a symbol for appreciation to the students is an important subject that has been taken for granted. Sacrifices made by nurses in the Iran-Iraq war played a significant role in redefining nursing and participation of nurses in the community; however, such activities have not yet been properly clarified. Moreover, no prominent figure in nursing has yet been introduced to the Iranian society. There is a deeply felt need for planning the identification and introduction of pioneers and prominent figures in nursing. Hader (2013) writes of appreciation, “the word ‘Thank you’ is a humble statement that contains a powerful message that is difficult to estimate.

In the present study, conditions such as, unequal opportunities of clinical growth for physicians and nurses and biased attention to medical schools were cited as other deficiencies in nursing education. Such factors have reduced opportunities for the presence of nurses in decision-making domains, in particular decision-makings pertaining to care. Dinmohammadi, Hushmand, Cheraghi, and Parovi (2014) regard these conditions as the product of the domineering/dominated phenomenon, and believe it to be the result of social norms and accepted laws and assumptions. The dominated group is comprised of people with limited capacities, such as in their social status, power, independence and access to resources, in comparison with the domineering group. Hughes (2001) also argues that it is possible to create equal opportunities for the participation of all groups in the health system; however, organizational systems divert the path of participation by creating unequal ranks in the Ministry of Health or through the unequal allocation of resources and provide a different perspective on healthcare which often halted by intense efforts on the part of physicians to preserve their monopoly on opinions of health matters. The present study also considered the instrumental, non-academic view of nursing as another deficiency in nursing education. Toofany (2005) writes of this issue, “decision-making in the National Health System should be based on credible research data, but due to the non-academic perspective on the discipline of nursing, progressive research and strategies proposed by nurses are much less noted, thus disenchanting nurses with participation in decision-making processes.

## 5. Conclusions

This study presented a fresh perspective on nursing education in Iran. Educating nurses who can challenge community health matters with their different academic levels and care capabilities is a serious requirement of nursing in the Iranian society at the present moment. In order to achieve this level of participation from the side of nursing graduators we need for revising and directing nursing education toward service learning, community based need programs such as diabetes and driving accidents and also totally application of present educational opportunities. The specialization of nursing and the establishment of specialized nursing associations, the emphasis on teaching the science of care and reinforcing the sense of appreciation of pioneers of nursing in Iran are among the directions offered in the present study. Results of this study provide a clear direction for policymakers in the field of nursing education and also for nursing instructors, particularly at the PhD level, to plan proper use of the available chances in the doctrine of nursing in order to train nurses willing to participate in decision-making processes. As in all other qualitative studies, results of the present study rely on the examined context and any generalization of the findings should be conducted with care.

## References

[1] Hajbaghery M. A, Salsali M (2005). A model for empowerment of nursing in Iran. BMC health services research.

[2] Arabi A, Rafii F, Cheraghi M. A, Ghiyasvandian S (2014). Nurses’ policy influence: A concept analysis. Iranian journal of nursing and midwifery research.

[3] Benton D (2012). Advocating globally to shape policy and strengthen nursing influence. OJIN: The Online Journal of Issues in Nursing.

[4] Boswell C, Cannon S, Miller J (2005). Nurses’ political involvement: Responsibility versus privilege. Journal of Professional Nursing.

[5] Braun V, Clarke V (2006). Using thematic analysis in psychology. Qualitative research in psychology.

[6] Buerhaus P. I, Needleman J, Mattke S, Stewart M (2002). Strengthening hospital nursing. Health Affairs.

[7] Byrd M. E, Costello J, Gremel K, Schwager J, Blanchette L, Malloy T. E (2000). Political astuteness of baccalaureate nursing students following an active learning experience in health policy. Public Health Nursing.

[8] Canadian Nurses Association (2000). Nursing is a political act: The bigger picture. Nursing Now: Issues and Trends in Canadian Nursing.

[9] Cheraghi M.-A, Jasper M, Vaismoradi M (2014). Clinical nurses’ perceptions and expectations of the role of doctorally-prepared nurses: A qualitative study in Iran. Nurse education in practice.

[10] Cheraghi M. A, Salasli M, Ahmadi F (2007). Iranian nurses’ perceptions of theoretical knowledge transfer into clinical practice: A grounded theory approach. Nursing & health sciences.

[11] Cheraghi M. A, Salsali M, Safari M (2010). Ambiguity in knowledge transfer: the role of theory-practice gap. Iranian journal of nursing and midwifery research.

[12] Cohen S. S, Milone-Nuzzo P (2001). Advancing health policy in nursing education through service learning. Advances in Nursing Science.

[13] Conger C. O. N, Johnson P (2000). Integrating political involvement and nursing education. Nurse Educator.

[14] Dinmohammadi M, Peyrovi H, Mehrdad N (2014a). Concept analysis of professional socialization in nursing. Nursing forum.

[15] Dinmohammadi M. R, Hushmand A, Cheraghi M. A, Peyrovi H (2014b). Oppression in Nursing Profession and The Way of its Management. Hospital Journal.

[16] Emerson R. J (2007). Nursing education in the clinical setting.

[17] Ferguson S. L (2001). An activist looks at nursing’s role in health policy development. Journal of Obstetric, Gynecologic, & Neonatal Nursing.

[18] Hader R, Ne-Bc R. N, Officer C. N The power of appreciation. Nursing Management.

[19] Hughes F. A (2005). Editorial. Policy a practical tool for nurses and nursing. Journal of Advanced Nursing.

[20] Hughes F. A (2002). Role of the government chief nurse in policy and the profession. Nursing and Health Policy Review.

[21] Hewison A (2008). Evidence-Based Policy Implications for Nursing and Policy Involvement. Policy, Politics, & Nursing Practice.

[22] Jasper P. M (2007). Editorial. Life at work modernizing nursing careers. Journal of nursing management.

[23] Keith J (2000). It takes two hands to make a sound. Nursing New Zealand (Wellington, NZ, 1995).

[24] Mason D. J, Leavitt J. K, Chaffee M. W (2011). Policy and Politics in Nursing and Healthcare-Revised Reprint.

[25] Milstead J. A (2008). Health policy and politics: a nurse’s guide.

[26] Ressler P, Glazer G (2011). Legislative: Nursing engagement in health policy and healthcare through social media. The Online Journal of Issues in Nursing.

[27] Reutter L, Duncan S (2002). Preparing nurses to promote health-enhancing public policies. Policy, Politics, & Nursing Practice.

[28] Russell K. A (2012). Nurse practice acts guide and govern nursing practice. Journal of Nursing Regulation.

[29] Sandelowski M (2010). What’s in a name? Qualitative description revisited. Research in nursing & health.

[30] Speziale H. S, Streubert H. J, Carpenter D. R (2007). Qualitative research in nursing: Advancing the humanistic imperative.

[31] Swanson M. L, Stanton M. P (2013). Chief Nursing Officers’ Perceptions of the Doctorate of Nursing Practice Degree. Nursing forum.

[32] Toofany S (2005). Nurses and health policy: Swaleh Toofany asks how nurses can influence health policy in the new political environment following the election. Nursing Management.

[33] University of Nevada, Las Vegas, school of nursing (2011-2012). PhD in nursing program. student handbook.

[34] Vaismoradi M, Parsa Yekta Z (2011). Iranian nursing students comprehension and experiences regarding evaluation process: A thematic analysis study. Scandinavian journal of caring sciences.

[35] Vaismoradi M, Turunen H, Bondas T (2013). Content analysis and thematic analysis: Implications for conducting a qualitative descriptive study. Nursing & health sciences.

[36] Villeneuve M. J (2008). Yes we can!Eliminating health disparities as part of the core business of nursing on a global level. Policy, Politics, & Nursing Practice.

[37] Zauderer C. R, Ballestas H. C, Cardoza M. P, Hood P, Neville S. M (2007). United we stand: preparing nursing students for political activism. The Journal of the New York State Nurses’ Association.

